# Efficacy and safety of NOAC versus warfarin in AF patients with left atrial enlargement

**DOI:** 10.1371/journal.pone.0243866

**Published:** 2020-12-14

**Authors:** Victor Chien-Chia Wu, Chun-Li Wang, Shu-Ting Gan, Michael Wu, Shao-Wei Chen, Chang-Fu Kuo, Yu-Tung Huang, Ming-Shien Wen, Shang-Hung Chang

**Affiliations:** 1 Division of Cardiology, Chang Gung Memorial Hospital, Linkou Medical Center, Taoyuan City, Taiwan; 2 College of Medicine, Chang Gung University, Taoyuan City, Taiwan; 3 Center for Big Data Analytics and Statistics, Chang Gung Memorial Hospital, Linkou Medical Center, Taoyuan City, Taiwan; 4 Divison of Cardiovascular Medicine, Arrhythmia Services Section, Rhode Island Hospital, Warren Alpert School of Medicine, Brown University, Providence, Rhode Island, United States of America; 5 Department of Cardiothoracic and Vascular Surgery, Chang Gung Memorial Hospital, Linkou Medical Center, Taoyuan City, Taiwan; 6 Division of Rheumatology, Allergy and Immunology, Department of Internal Medicine, Chang Gung Memorial Hospital, Linkou Medical Center, Taoyuan City, Taiwan; 7 Division of Rheumatology, Orthopaedics and Dermatology, School of Medicine, University of Nottingham, Nottingham, United Kingdom; 8 Graduate Institute of Nursing, Chang Gung University of Science and Technology, Taoyuan City, Taiwan; Maastricht University Medical Center, NETHERLANDS

## Abstract

**Background:**

Little is known about the effects of anticoagulation in patients with atrial fibrillation (AF) and left atrial enlargement (LAE).

**Methods:**

Data of patients with AF were retrieved from Chang Gung Research Database during 2007–2016. We excluded patients who were not using oral anticoagulants, used anticoagulants for <30 days, used ≥2 agents concomitantly or switched anticoagulants, had left atrial diameter missing from their data, were aged <65, had received valve surgeries, had mitral stenosis, or had a history of cancer. The primary outcomes were ischemic stroke (IS)/systemic embolism (SE), major bleeding, and death from any cause.

**Results:**

We identified 40,777 patients who received a diagnosis of AF. After the exclusion criteria were applied, 6,445 patients remained, 4,922 with LAE, and they were followed up for 2.4 ±1.9 years. The mean age of the patients was 77.32 ± 0.18 in the NOAC group and 76.58 ± 6.91 in the warfarin group (*p* < 0.0001); 48.24% of patients in the NOAC group and 46.98% of patients in the warfarin group were men (*p* > 0.05). The mean CHA_2_DS_2_-VASc score was 3.26 ± 1.05 in the NOAC group and 3.07 ± 1.12 in the warfarin group (*p* < 0.0001). The mean HAS-BLED score was 3.87 ± 3.81 in the NOAC group and 3.86 ± 3.80 in the warfarin group (*p* > 0.05). Furthermore, the mean LA diameter was 4.75 ± 0.63 cm in the warfarin group and 4.79 ± 0.69 cm in the warfarin group (*p* > 0.05). Among patients with LAE, NOAC was associated with significantly reduced IS/SE events (CRR = 0.63, 95% CI = 0.52–0.77), no difference in major bleeding (CRR = 0.91, 95% CI = 0.78–1.05), and significantly reduced death from any cause (aHR = 0.65, 95% CI = 0.52–0.80) compared with warfarin.

**Conclusions:**

In elderly patients with AF and LAE, NOAC was associated with reduced IS/SE and death from any cause compared with warfarin, whereas no difference in major bleeding was observed between these treatments.

## Introduction

Patients with atrial fibrillation (AF) are treated with anticoagulation for stroke prevention based on their CHA_2_DS_2_-VASc risk score [1,2]. However, the risk of ischemic stroke (IS) in these patients remains substantial even with the appropriate use of anticoagulation following the guideline recommendations, suggesting that other factors are at play. One critical contributor is left atrium size, which has been described as a risk factor for nonvalvular AF [3,4]. Increased left atrial size has been demonstrated to reflect the AF disease duration and burden [5,6]. Notably, studies have demonstrated that left atrial enlargement (LAE), independent of CHA_2_DS_2_-VASc score, is also a risk predictor for IS [7–10].

Despite the remarkable benefits of warfarin and novel vitamin K–antagonist oral anticoagulants (NOAC) in reducing stroke risk and mortality in patients with AF, the residual stroke risk was 1.7% and 1.4% per year among patients receiving warfarin and NOACs, respectively, at the end of a 2.2-year follow-up [11]. A study on therapeutic anticoagulation treatment for IS among patients with AF reported that moderate to severe LAE was associated with treatment failure [12]. Landmark trials on NOACs versus warfarin were conducted in patients with AF with high stroke risk based on CHAD2 score [13–16]. These studies did not conclude whether NOACs are associated with more favorable outcomes compared with warfarin in patients with AF marked by normal or enlarged left atrial (LA) size. Therefore, we investigated the efficacy and safety profiles of NOAC and warfarin in patients with AF with LAE.

## Methods

### Data source

In this retrospective cohort study, patient data were obtained from the Chang Gung Memorial Hospital System, which is the largest health care provider in Taiwan, comprising three major teaching hospitals and four tertiary-care medical centers [13–16]. This health care provider has over 10,000 beds and admits more than 280,000 patients, servicing approximately one-tenth of the Taiwanese population each year. The hospital identification number of each patient was encrypted and deidentified to protect their privacy. Informed consent was thus waived for this study. The diagnosis and laboratory data were linked and continuously monitored using consistent data encryption. The Institutional Review Board of Chang Gung Memorial Hospital approved the study protocol (IRB No. 201802093B0).

### Data availability statement

The data are owned by Chang Gung Memorial Hospital and were obtained for analysis from the Chang Gung Research Database (CGRD). Data in the CGRD can be accessed by contacting the Chang Gung Memorial Hospital Center for Big Data Analytics and Statistics (https://www1.cgmh.org.tw/rccr/). Therefore, researchers can replicate the study findings in their entirety by directly obtaining the data from CGRD and following the protocol presented in the Methods section. The authors of this study do not have any special access privileges.

### Study patients

A search of the electronic medical records of the CGRD between January 1, 2007, and December 31, 2016, yielded data from patients with a diagnosis of AF based on at least one inpatient or two outpatient claims for nonvalvular AF. Patients who were not receiving oral anticoagulants (e.g., warfarin, dabigatran, rivaroxaban, apixaban, or edoxaban) or had an anticoagulation use <30 days, had concomitant use of ≥2 agents, switched the oral anticoagulant used, had missing LA diameter data, had a history of valve surgery, had mitral stenosis, or had a history of cancer were excluded. Furthermore, patients aged <65 years were excluded because Taiwan’s National Health Insurance only reimburses NOAC prescriptions for patients aged ≥65 years. The included patients, diagnosed as having AF and using oral anticoagulants, were separated into patients with normal LA size and patients with LAE.

### Definition of LAE

Based on the 2015 American Society of Echocardiography guidelines on Recommendations for Cardiac Chamber Quantification in Adults, normal anteroposterior LA diameters are 2.7–3.8 cm in women and 3.0–4.0 cm in men [17]. Therefore, LAE was defined as anteroposterior diameters of >3.8 cm in women and >4.0 cm in men in this study.

### Study outcomes and follow-up

Primary outcomes were defined as IS/systemic embolism (SE), major bleeding, and death from any cause at the end of follow-up [18]. Major bleeding was defined based on principal or secondary diagnosis at hospitalizations and emergency visits and any blood transfusion order, including admission for any bleeding, a need for a blood transfusion of >2 U, and life-threatening bleeding or vital organ hemorrhage (e.g., intracerebral hemorrhage). The follow-up period was defined as the period from the index date until the first occurrence of any study outcome or the end date of the study period (December 31, 2016), whichever came first.

We applied the *International Classification of Diseases*, *Ninth Revision*, *Clinical Modification* (ICD-9-CM) and ICD-10 codes to categorize diseases. Covariates included age, sex, CHA_2_DS_2_-VASc score, HAS-BLED score, LA size, comorbidities, and medications. The comorbidities included were diabetes mellitus, hypertension, heart failure, renal insufficiency, peptic ulcer disease, abnormal liver function, peripheral artery disease, and old myocardial infarction. The medications included were antiplatelets, angiotensin-converting enzyme inhibitors/angiotensin receptor blockers, amiodarone/dronedarone, beta blockers, calcium channel blockers, diuretics, NSAIDs, and antidiabetic drugs.

### Statistical analysis

The *t* test was used to assess continuous variables, and the χ^2^ test was used to assess categorical variables. The propensity score was included as a covariate based on the CHA_2_DS_2_-VASc and HAS-BLED scores. The risk of death from any cause was compared between groups using a Cox proportional hazards model. Competing risk regression (CRR) was performed with IS/SE and major bleeding. A *p* value <0.05 was considered statistically significant. No adjustments for multiple testing (multiplicity) were used in this study. All statistical analyses were performed using SAS 9.4 (SAS Institute, Cary, NC).

### Sensitivity analysis

Two sensitivity analyses were performed to validate our study findings and assess selection biases. In the first analysis, patients who were not using oral anticoagulants, such as warfarin, dabigatran, rivaroxaban, apixaban, or edoxaban, or had not used anticoagulants for <90 days were excluded; the other exclusion criteria remained the same. In the second analysis, outcome analyses were performed based on left atrial diameter (LAD) indexed to body surface area (BSA). A study reported that the LAD index value of normal control was 20 ± 3 mm/m^2^ (mean ± SD) [19]. The LAD index > 26 mm/m^2^ indicated dilated when 26 mm/m^2^ (mean + 2SD) was used as the cutoff value.

## Results

### Study population

**Fig 1:**
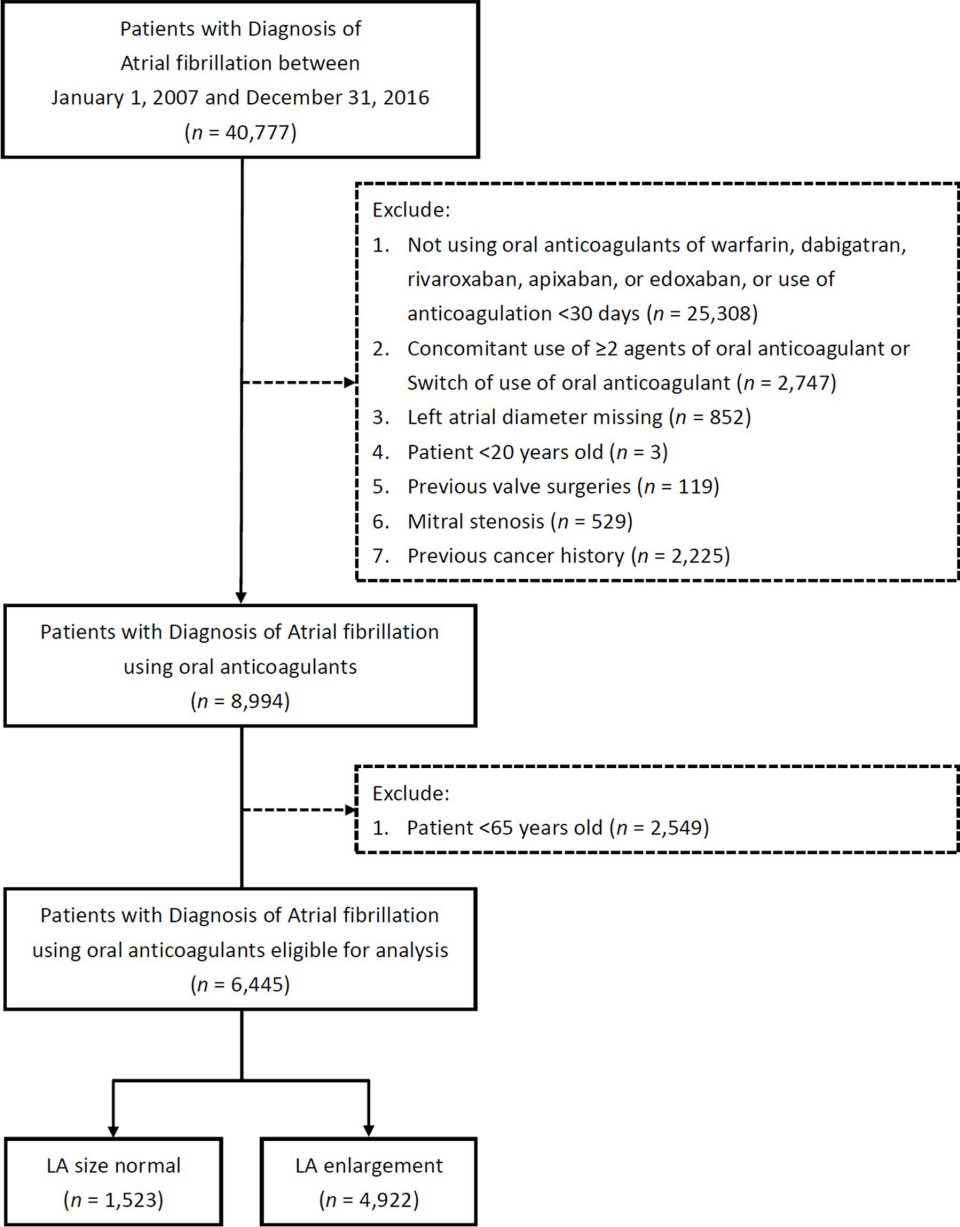
Study design and screening criteria flowchart for the inclusion of elderly AF patients with LAE. AF, atrial fibrillation; LA, left atrial enlargement.

We identified 40,777 patients with a principal diagnosis of AF during 2007–2016 in the CGRD. After exclusion criteria, a group of 6,445 patients remained. LAE was present in a total of 4,922 patients that comprise the study population (**Fig 1 and Table 1**). The mean follow-up time was 2.4 ± 1.9 years. The time in therapeutic range was assessed based on an international normalized ratio between 2.0 and 3.0, which revealed that the warfarin therapy quality was 44.81% in the study patients. The mean age of the patients was 77.32 ± 0.18 in the NOAC group and 76.58 ± 6.91 in the warfarin group (*p* < 0.0001), with 48.24% men in the NOAC group and 46.98% men in the warfarin group (*p* = ns). The mean CHA_2_DS_2_-VASc score was 3.26 ± 1.05 in the NOAC group and 3.07 ± 1.12 in the warfarin group (*p* < 0.0001). The mean HAS-BLED score was 3.87 ± 3.81 in the NOAC group and 3.86 ± 3.80 in the warfarin group (*p* = ns). Furthermore, LA diameter was 4.76 ± 0.63 cm in the NOAC group and 4.79 ± 0.69 cm in the warfarin group (*p* = ns). Overall, patients in the NOAC group were significantly older and had significantly higher CHA_2_DS_2_-VASc scores, reflecting a high risk of stroke.

**Table 1:** Baseline characteristics of study patients.

	LAE				
	NOAC (n = 1,592)		Warfarin (n = 1,592)		Test
	Median	IQR	Median	IQR	*p*-value*
Age	77	72–83	76	71–81	0.009
CHA_2_DS_2_-VASc score	3	3–4	3	2–4	<0.001
	Mean	SD	Mean	SD	*p*-value^†^
HAS-BLED score	3.87	1.21	3.86	1.19	0.859
LA size	4.76	0.63	4.79	0.69	0.185
	N	%	N	%	*p*-value^‡^
Male	768	48.24	748	46.98	0.478
Comorbidities					
Diabetes mellitus	555	34.86	524	32.91	0.246
Hypertension	1302	81.78	1101	69.16	< .0001
Heart failure	729	45.79	750	47.11	0.456
Renal insufficiency	496	31.16	506	31.78	0.703
Peptic ulcer disease	283	17.78	286	17.96	0.890
Abnormal liver function	164	10.30	173	10.87	0.604
Peripheral artery disease	23	1.44	45	2.83	0.007
Old myocardial infarction	103	6.47	122	7.66	0.189
Medications					
Antiplatelets	458	28.77	452	28.39	0.814
ACEi/ARB	486	30.53	492	30.90	0.818
Amiodarone/dronedarone	513	32.22	498	31.28	0.568
Beta blockers	454	28.52	477	29.96	0.370
Calcium channel blockers	308	19.35	296	18.59	0.588
Diuretics	399	25.06	415	26.07	0.516
NSAIDs	169	10.62	175	10.99	0.732
Antidiabetic drug	217	13.63	231	14.51	0.476

IQR, interquartile range; SD, standard deviation.

* Wilcoxon rank-sum test.

^†^ independent t test.

^‡^ χ^2^ test.

### Primary outcomes

**Fig 2:**
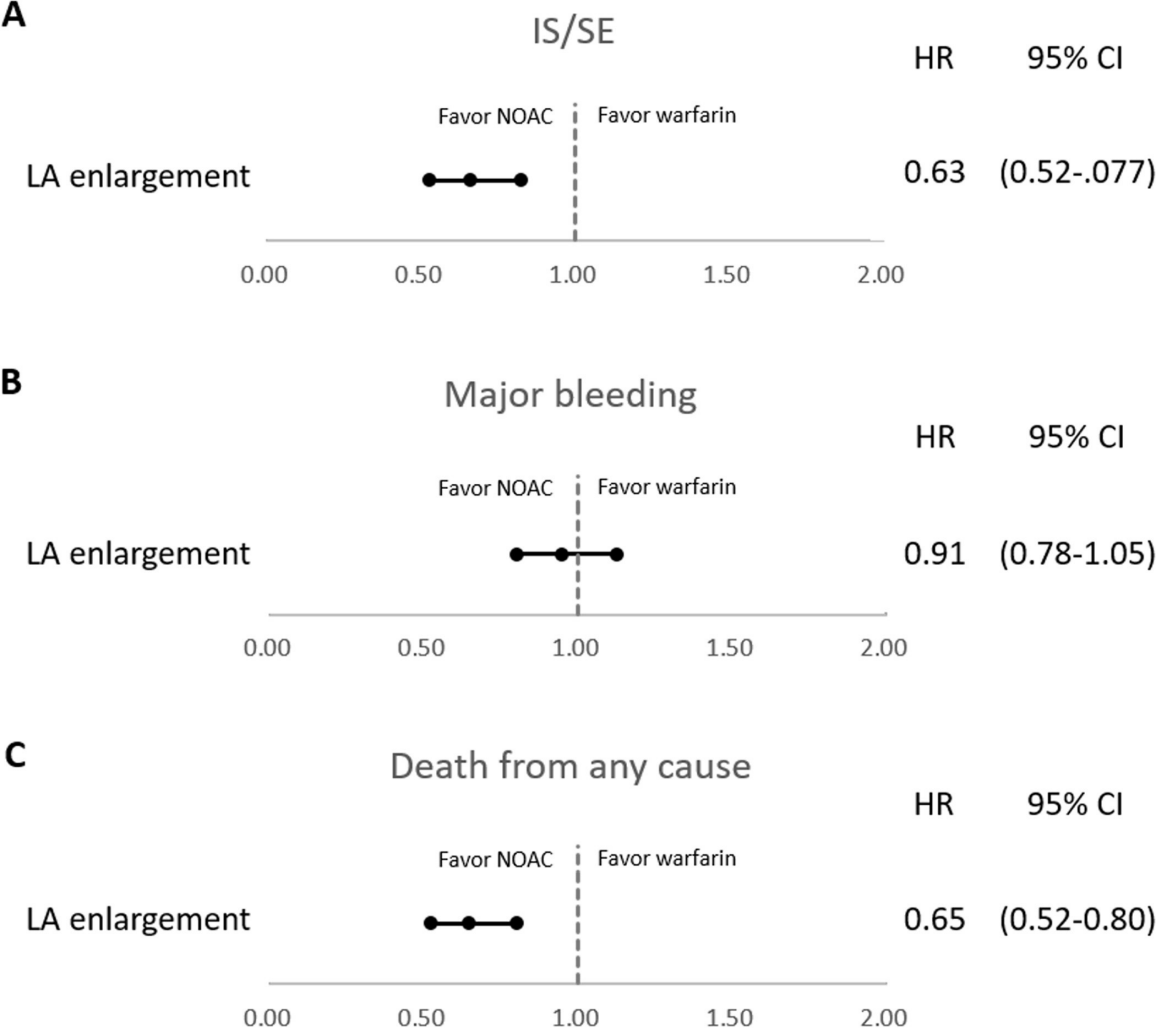
Primary outcomes that occurred during follow-up. (A) IS/SE, (B) major bleeding, (C) death from any cause. CI, confidence interval; HR, hazard ratio; IS/SE, ischemic stroke/systemic embolism.

**Fig 3:**
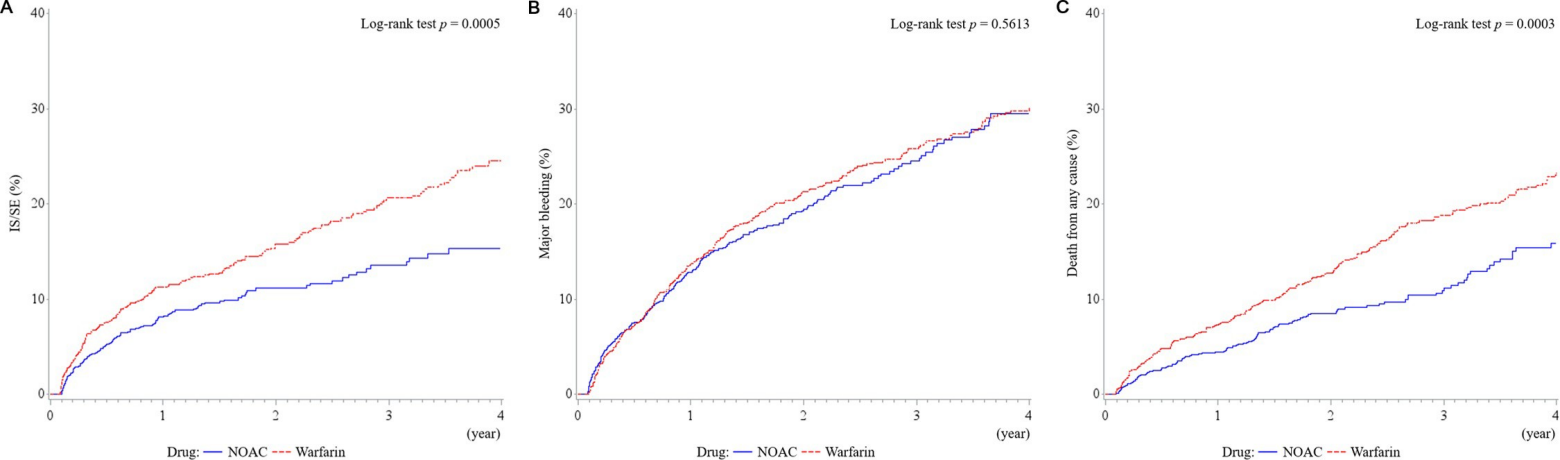
Cumulative incidence of IS/SE (A), major bleeding (B), and Kaplan–Meier survival analysis of death from any cause (C) in elderly patients with AF with LAE treated with NOAC and warfarin.

In patients with LAE, patients receiving NOAC had a significantly lower number of IS/SE events (CRR: 0.63, 95% CI: 0.52–0.77, *p* < 0.001) **(Table 2 and Fig 2)** at the end of follow-up compared with patients receiving warfarin. Cumulative incidence analysis revealed a significantly lower number of IS/SE events in the NOAC group (*p* = 0.0005) **(Fig 3A)**. Patients receiving NOAC displayed no difference in major bleeding compared with patients receiving warfarin (CRR: 0.91, 95% CI: 0.78–1.05, *p* = 0.190). Cumulative incidence analysis revealed no difference in major bleeding between the groups (*p* = 0.5613) **(Fig 3B)**. Furthermore, compared with patients receiving warfarin, patients receiving NOAC had significantly reduced death from any cause (adjusted hazard ratio [aHR]: 0.65, 95% CI: 0.52–0.80, *p* < 0.001). Kaplan–Meier survival analysis revealed significantly reduced death from any cause in the NOAC group (*p* = 0.0003) **(Fig 3C)**. Based on our major findings, we calculated the observed (post hoc) power to be as follows: IS/SE, 0.82; major bleeding, 0.08; and death from any cause, 0.84.

**Table 2:** Hazard ratios for outcomes in patients with LAE.

Ischemic stroke/Systemic embolism						
Drug	Patients	Events	Incidence	Crude HR	Adjusted HR	Competing Risk HR
NOAC	1,253	130	10.38	0.67 (0.54–0.84)*	0.66 (0.53–0.83)*	0.63 (0.52-.77)*
Warfarin	1,161	209	18.00	1	1	1
Major bleeding						
Drug	Patients	Events	Incidence	Crude HR	Adjusted HR	
NOAC	1,378	255	18.51	0.95 (0.80–1.13)	0.95 (0.80–1.12)	0.91 (0.78–1.05)
Warfarin	1,348	325	24.11	1	1	
Death from any cause						
Drug	Patients	Events	Incidence	Crude HR	Adjusted HR	
NOAC	1,466	134	9.14	0.67 (0.54–0.83)*	0.65 (0.52–0.80)*	
Warfarin	1,425	278	19.51	1	1	

Model adjusted for CHA_2_DS_2_-VASc and HAS-BLED scores; *p* < 0.05.

HR, hazard ratio; LA, left atrial; NOAC, novel vitamin K–antagonist oral anticoagulant.

### Sensitivity analysis

The first sensitivity analysis indicated that patients receiving NOAC had significantly reduced IS/SE at the end of follow-up compared with patients receiving warfarin (CRR: 0.60, 95% CI: 0.48–0.76) **(S1 Table)**. Patients receiving NOAC displayed no difference in major bleeding compared with patients receiving warfarin (CRR: 0.86, 95% CI: 0.73–1.02), but they displayed significantly reduced death from any cause (aHR: 0.68, 95% CI: 0.54–0.85).

In the second sensitivity analysis, a LAD index > 26 mm/m^2^ was considered dilated. After excluding patients with missing BSA data, data from 1,430 patients were analyzed (NOAC group: 749 patients, warfarin group: 681 patients). Patients with a dilated LAD index receiving NOAC displayed significantly reduced IS/SE at the end of follow-up compared with those receiving warfarin (CRR: 0.61, 95% CI: 0.41–0.90) **(S1 Table)**. Patients receiving NOAC had no difference in major bleeding compared with patients receiving warfarin (CRR: 0.90, 95% CI: 0.69–1.18). Furthermore, death from any cause was significantly reduced among patients receiving NOAC compared with patients receiving warfarin (aHR: 0.69, 95% CI: 0.51–0.95).

## Discussion

To our knowledge, this is the first study to investigate and compare the outcomes of NOAC compared with warfarin in elderly patients with AF aged ≥65 years with normal and enlarged LA sizes; key findings are as follows: Patients with LAE receiving NOAC were associated with significantly reduced IS/SE events and deaths from any cause, without differences in major bleeding compared with patients with LAE receiving warfarin.

The risk of thromboembolism, resulting in stroke and systemic arterial occlusion, in patients with AF is a major concern, and treatment guidelines are continually updated to offer more precise approaches to predicting and preventing these events [20–22]. The CHA_2_DS_2_-VASc score was developed as an improvement on the CHAD_2_ score for more accurate risk prediction and anticoagulation guidance [23]. Landmark trials of NOACs versus warfarin were based on risk assessment by using CHAD_2_ score [24–27]. However, few AF guidelines have addressed the effect of LAE on stroke risk or accounted for AF in stroke risk score calculation. Furthermore, LA size was not mentioned in the landmark trials, even though LAE was a clear marker of AF disease status and a predictor of stroke risk [3–10].

The mechanisms of the increased risk of stroke in patients with LAE remains poorly understood. However, left atrium dilation is associated with the loss of atrial pump function and an increase in blood stasis, which in turn predispose the patient to LA thrombus formation and embolization [28]. The thrombogenicity of LA dilation was also confirmed in transesophageal echocardiography studies that reported that LAE was associated with increased spontaneous echo contrast, LA thrombus formation, and embolic events [29]. Thus, these results suggest a potential causal relationship between LAE and subsequent thromboembolism and stroke.

A study to determine the residual stroke rate, which is often regarded as treatment failure, compared patients with AF receiving anticoagulation treatment with a matched control population with comparable baseline risks and without AF [30]. The cumulative mortality at 1.5 years was 4.2% in patients with AF receiving warfarin and 2.5% in the matched controls, even after adjustment for baseline differences (*p* = 0.005) [30]. This residual mortality risk may be attributed to additional AF stroke risk factors that were not accounted for in the traditional CHA_2_DS_2_-VASc scores, such as LA size [7–10]. Furthermore, reduced LA size was demonstrated to have protective effects against AF and AF-associated complications by reducing the inducibility and duration of AF [31].

In this study, we investigated whether the residual stroke risk associated with LAE was more improved by taking NOAC rather than warfarin in terms of efficacy, safety, and mortality profiles. Elderly patients with AF (≥65 years old) with LAE were studied. Our results revealed that NOAC had consistent benefits over warfarin, with significantly reduced IS/SE and death from any cause in patients with LAE or LAD index dilatation. Furthermore, no difference in major bleeding was observed between patients with LAE or LAD index dilatation taking NOAC or warfarin. Anticoagulation treatment failure has been encountered in patients with AF with marked LAE [12], and our study revealed that NOAC may confer mortality benefits compared with warfarin in patients with LAE. In the first sensitivity analysis, we excluded patients who had treatment times with anticoagulants of <90 days, and the results were similar to the main study, which excluded patients with treatment times with anticoagulants of <30 days. In the second sensitivity analysis, in which we performed outcome analysis based on the LAD index, the results were also similar to the main study. The strength of the current study provides important real-world data that confirm the superiority of NOACs compared to warfarin to reduce the risk for stroke in patients with AF.

In summary, compared with warfarin, the use of NOAC was associated with reduced IS/SE and mortality events in elderly patients with AF and LAE. Further studies are warranted to clarify the role of LAE among current AF stroke risk scores.

### Limitations

Epidemiologic data from the CGRD have several limitations. First, the use of ICD-9-CM and ICD-10 codes may lead to missing cases of patient conditions that were incorrectly coded. Second, we did not analyze individual NOACs to delineate the efficacy and safety of each drug compared with warfarin because the number of recently introduced edoxaban users was relatively small. Third, we only included patients aged ≥ 65 years because Taiwan’s National Health Insurance does not reimburse the NOAC prescriptions of patients aged <65 years, which may have resulted in selection bias. However, most patients develop AF in older age. Therefore, our study likely still represents the majority of patients with AF in Taiwan. Fourth, the data retrieved from the CGRD represent patients with more severe disease status than patients from regional hospitals or local clinics because these data were obtained from teaching hospitals and tertiary medical centers within the Chang Gung Memorial Hospital System. Fifth, since the current study has a non-randomized retrospective design, the results should be treated with a certain caution and should be considered hypothesis generating. Last, this study was conducted in an ethnically homogenous population; therefore, the application of the results in other populations may require further studies.

## Conclusions

In elderly AF patients ≥65 years old with LAE, using NOAC was associated with reduced ischemic stroke/systemic embolism events as well as deaths from any cause compared with using warfarin, with no differences of major bleeding events.

## Supporting information

S1 Table Hazard ratios for outcomes in patients with LAE (follow-up > 90 days). (DOCX)

S2 Table Hazard ratios for outcomes in patients with dilated LAD indexed to BSA (>26 mm/m^2^). (DOCX)
