# Supplementary material for: Efficacy and safety of NOAC versus warfarin in AF patients with left atrial enlargement
Source: PLoS One. 2020 Dec 14;15(12):e0243866. doi: 10.1371/journal.pone.0243866 (PMC7735599; doi:10.1371/journal.pone.0243866)
Supplement: S1 Table — (DOCX) [file pone.0243866.s001.docx]

**S1 Table.** Hazard ratios for outcomes in patients with LAE (follow-up > 90 days)

Ischemic stroke/Systemic embolism

| Drug | Patients | Events | Incidence | Crude HR | Adjusted HR | Competing Risk HR |
| --- | --- | --- | --- | --- | --- | --- |
| NOAC | 1,148 | 94 | 8.19 | 0.67 (0.52-0.87)* | 0.65 (0.50-0.85)* | 0.60 (0.48-0.76)* |
| Warfarin | 1,020 | 161 | 15.78 | 1 | 1 | 1 |

Major bleeding

| Drug | Patients | Events | Incidence | Crude HR | Adjusted HR |  |
| --- | --- | --- | --- | --- | --- | --- |
| NOAC | 1,245 | 192 | 15.42 | 0.90 (0.75-1.09) | 0.89 (0.74-1.08) | 0.86 (0.73-1.02) |
| Warfarin | 1,192 | 272 | 22.82 | 1 | 1 |  |

Death from any cause

| Drug | Patients | Events | Incidence | Crude HR | Adjusted HR |  |
| --- | --- | --- | --- | --- | --- | --- |
| NOAC | 1,379 | 115 | 8.34 | 0.71 (0.56-0.90)* | 0.68 (0.54-0.85)* |  |
| Warfarin | 1,301 | 242 | 18.60 | 1 | 1 |  |

Model adjusted for CHA_2_DS_2_-VASc and HAS-BLED scores; *p* < 0.05.

HR, hazard ratio; LA, left atrial; NOAC, novel vitamin K–antagonist oral anticoagulant.
