# Supplementary material for: Efficacy and safety of NOAC versus warfarin in AF patients with left atrial enlargement
Source: PLoS One. 2020 Dec 14;15(12):e0243866. doi: 10.1371/journal.pone.0243866 (PMC7735599; doi:10.1371/journal.pone.0243866)
Supplement: S2 Table — (DOCX) [file pone.0243866.s002.docx]

**S2 Table.** Hazard ratios for outcomes in patients with dilated LAD indexed to BSA (>26 mm/m^2^)

Ischemic stroke/Systemic embolism

| Drug | Patients | Events | Incidence | Crude HR | Adjusted HR | Competing Risk HR |
| --- | --- | --- | --- | --- | --- | --- |
| NOAC | 548 | 67 | 12.23 | 0.78 (0.57-1.08)* | 0.78 (0.57-1.08)* | 0.61(0.41-0.90)* |
| Warfarin | 618 | 97 | 15.70 | 1 | 1 | 1 |

Major bleeding

| Drug | Patients | Events | Incidence | Crude HR | Adjusted HR |  |
| --- | --- | --- | --- | --- | --- | --- |
| NOAC | 669 | 131 | 19.58 | 0.95 (0.75-1.21) | 0.97 (0.76-1.23) | 0.90 (0.69-1.18) |
| Warfarin | 600 | 163 | 27.17 | 1 | 1 |  |

Death from any cause

| Drug | Patients | Events | Incidence | Crude HR | Adjusted HR |  |
| --- | --- | --- | --- | --- | --- | --- |
| NOAC | 711 | 66 | 9.28 | 0.69 (0.51-0.94)* | 0.69 (0.51-0.95)* |  |
| Warfarin | 635 | 130 | 20.47 | 1 | 1 |  |

Model adjusted for CHA_2_DS_2_-VASc and HAS-BLED scores; *p* < 0.05.

HR, hazard ratio; LAD, left atrial diameter; NOAC, novel vitamin K–antagonist oral anticoagulant.
